# Dyspepsia: When and How to Test for* Helicobacter pylori* Infection

**DOI:** 10.1155/2016/8463614

**Published:** 2016-04-28

**Authors:** Maria Pina Dore, Giovanni Mario Pes, Gabrio Bassotti, Paolo Usai-Satta

**Affiliations:** ^1^Dipartimento di Medicina Clinica e Sperimentale, Clinica Medica, University of Sassari, Viale San Pietro, No. 8, 07100 Sassari, Italy; ^2^Dipartimento di Medicina, Sezione di Gastroenterologia, University of Perugia, Piazza Lucio Severi 1, San Sisto, 06132 Perugia, Italy; ^3^Gastrointestinal Unit, P. Brotzu Hospital, 09124 Cagliari, Italy

## Abstract

Dyspepsia is defined as symptoms related to the upper gastrointestinal tract. Approximately 25% of western populations complain of dyspeptic symptoms each year. 70% of them do not have an organic cause and symptoms are related to the so-called functional dyspepsia, characterized by epigastric pain, early satiety, and/or fullness during or after a meal occurring at least weekly and for at least 6 months according to ROME III criteria. In order to avoid invasive procedures and adverse effects, to minimize costs, to speed up diagnosis, and to provide the most appropriate treatments, primary care physicians need to recognize functional dyspepsia. Because symptoms do not reliably discriminate between organic and functional forms of the disease, anamnesis, family history of peptic ulcer and/or of gastric cancer, medication history, especially for nonsteroidal anti-inflammatory drugs, age, and physical examination could help the physician in discerning between functional dyspepsia and organic causes. For patients without alarm symptoms, noninvasive testing for* H. pylori*, with either carbon-13-labeled urea breath testing or stool antigen testing, is recommended as a first-line strategy. In this review, we provide recommendations to guide primary care physicians for appropriate use of diagnostic tests and for* H. pylori* management in dyspeptic patients.

## 1. Introduction

Dyspepsia is defined as symptoms related to the upper gastrointestinal tract. With approximately 25% of western populations experiencing dyspepsia each year, dyspepsia is one of the most common causes for consulting a physician for a gastrointestinal complaint [1, 2]. Dyspeptic symptoms have been clustered into 3 categories:* ulcer-like dyspepsia *in which the predominant symptom is pain centered in the upper abdomen (most bothersome);* dysmotility-like dyspepsia*, an unpleasant or troublesome discomfort centered in the upper abdomen associated with upper abdominal fullness, early satiety, bloating, or nausea; and* unspecified* (*nonspecific*)* dyspepsia *defined as the presence of symptoms that do not fulfill the criteria for ulcer-like or dysmotility-like dyspepsia [3]. The most recent ROME III definitions exclude those patients with traditional heartburn and include duration of being symptomatic for 6 months prior to diagnosis and being an active problem for the last 3 months [1].

Dyspepsia is not a disease but rather is a symptoms complex associated with a wide spectrum of diseases. In most cases, no currently diagnosable organic disease is found and the problem is considered functional or idiopathic. The most common symptoms in patients with functional dyspepsia are (i) early satiation (inability to finish a normal sized meal), (ii) epigastric pain or burning (classified as the epigastric pain syndrome), and (iii) postprandial fullness (early satiation classified as the postprandial distress syndrome) [1]. However, because dyspepsia is a common presenting symptom of serious conditions such as peptic ulcer and gastric cancer, it is important that clinicians be able to stratify patients with dyspepsia with regard to the risk of the symptoms being related to a serious condition. This requires a logical approach to diagnosis and management. Until completion of the diagnostic assessment, all patients are characterized as having uninvestigated dyspepsia.

## 2. Evaluation of Uninvestigated Dyspepsia

Classically, the evaluation starts with a history and physical examination designed to separate organic and functional causes. Here, one searches for the presence of symptoms and findings suggestive of an organic disease (e.g., the so-called alarm symptoms or features) [2].

Alarm or “red flags” prompting endoscopy for the evaluation of patients with dyspepsia are as follows:

- Overt gastrointestinal bleeding.
- Anemia.
- Unexplained weight loss.
- Progressive dysphagia.
- Odynophagia.
- Recurrent vomiting.
- Family history of GI cancer.
- Presence of an abdominal mass and/or lymphadenopathy.

Overall, most alarm symptoms have a low predictive value for the presence of an organic disease [4, 5]. However, their presence would point toward early use of more invasive diagnostic maneuvers such as upper gastrointestinal endoscopy whereas absence of alarm features in a young, otherwise healthy individual would point toward an initial trial of medical therapy. The most feared diagnosis is gastric cancer and in regions with a high incidence of gastric cancer such as Japan or Korea the age of 45 is the cut-off. Where gastric cancer is not common, upper gastrointestinal endoscopy is recommended for patients above 55 years old [2, 3, 6, 7].

Among patients with dyspepsia, only 25% have an organic cause (8); in the rest of the patients, a diagnosis of functional dyspepsia can be made according to ROME III criteria (1).

## 3. *Helicobacter pylori* Infection and Dyspepsia

Although* H. pylori-*associated diseases commonly present with dyspepsia (e.g., peptic ulcer and gastric cancer), the infection itself may cause dyspepsia without obvious gross structural changes.* H. pylori* infection causes progressive functional and structural gastroduodenal damage that unpredictably may progress to peptic ulcer disease and its complications such as atrophic gastritis or gastric cancer as follows.

Clinical outcomes related to* Helicobacter pylori* infection are as follows:

 Active chronic gastritis:
   Impaired acid production.
   Impaired drug absorption.
   Atrophic gastritis.
   Impaired B12 vitamin absorption.
 Transmission of the infection to others especially family.
 Dyspepsia (nonulcer).
 Iron deficiency anaemia.
 Autoimmune thrombocytopenia.
 Peptic ulcer:
   Peptic ulcer complications.
 MALT lymphoma.
 Gastric adenocarcinoma.

Approximately 20% of those with an* H. pylori* infection will experience an* H. pylori*-related clinical disease [6]. Randomized controlled trials of* H. pylori* eradication therapy versus placebo report that only a proportion (10 to 12%) of functional dyspeptic patients achieve a significant improvement of persistent symptoms after* H. pylori* eradication [2, 8–12]. And relief may also take several months up to one year. A recent randomized clinical trial conducted in primary care patients with dyspeptic symptoms reported that 49% (94 of 192) improved compared to 36.5% (72 of 197) in the control group (*P* = 0.01; number needed to treat = 8). Similar results have been observed in dyspeptic patients from Asia [13, 14]. A population of* H. pylori* infected dyspeptic patients followed up for 7 years after* H. pylori* eradication showed a 25% reduction in consultations for dyspeptic symptoms [15]. Because eradication of* H. pylori* will eliminate dyspepsia in only a portion of infected dyspeptic patients, it is also important to know what to tell the patient about the short- and long-term expectations of* H. pylori* eradication. Overall, patients can be assured that cure of an* H. pylori* infection will result in healing of the gastritis and, depending on the reversibility of the damage that has occurred, return of function. Their risk of* H. pylori* peptic ulcers is eliminated and if ulcers are present, they will be cured. The risk of gastric cancer is also reduced and they can no longer transmit the infection to other family members [6]. Importantly, the effect on relief of dyspepsia is less assured [1, 2]. It is therefore important in the evaluation of dyspepsia to identify in which patients and when diagnostic tests for* H. pylori* should be done and which are the appropriate tests. Because it is not currently possible to identify which patient is at risk for a bad outcome, it has been recommended that all with* H. pylori* infections should receive* H. pylori* eradication therapy [7].

## 4. Approach for Patients in relation to Alarm Symptoms

**Figure 1:**
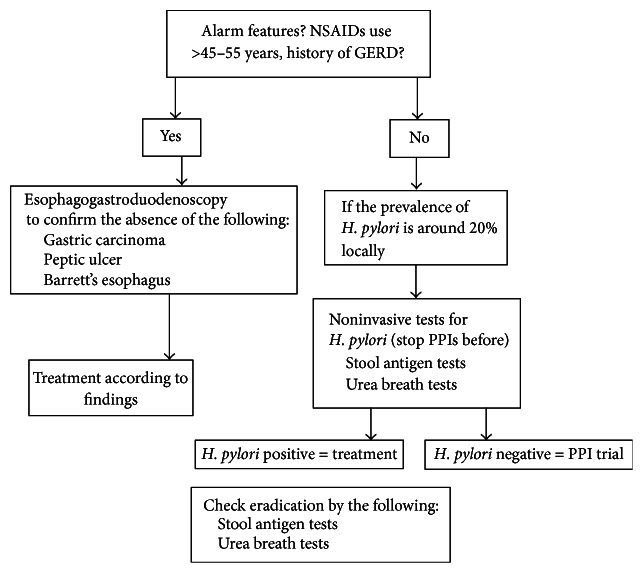
Flow chart of the management of* H. pylori* for dyspeptic patients with dyspepsia.

For patients with alarm features, early esophagogastroduodenoscopy is recommended (Figure 1). For those without alarm features, the decision is whether a trial of empiric proton pump inhibitor (PPI) therapy or further diagnostic testing. In areas where* H. pylori* infections are common (e.g., ≥20%), a test for* H. pylori* and treatment of infected individuals are preferred over a trial of therapy with PPIs. In such regions, the test-and-treat* H. pylori* strategy has proven cost-effective and decreases the number of endoscopies. However, to test for* H. pylori* as a first-line strategy is reasonable even in areas with low prevalence of infection, given that the available tests are not invasive. Studies on economic modeling and symptoms improvement suggest that eradication therapy is a cost-effective strategy for managing functional dyspepsia and more data demonstrated that the treatment is particularly effective for patients with peptic ulcer-like symptoms [1, 2, 16].

In those in whom dyspepsia remains despite* H. pylori* eradication, a trial of PPI therapy is a reasonable next step. If symptoms persist, treatment with a prokinetic agent, antidepressant drugs or some form of alternative medications, might be considered, although evidence from prospective studies to support this approach is limited [17].

## 5. Diagnostic Tests for* H. pylori* Infection

*H. pylori* infection is associated with a number of diseases (see the previous list of clinical outcomes related to* Helicobacter pylori* infection). There are many excellent tests currently available to identify active* H. pylori* infections as follows.

Noninvasive tests include the following:

 Serology:
   Blood IgG serology.
   Salivary assay.
   Urinary IgG assay.
 Urea breath test (UBT):
   13C-urea breath tests.
   14C-urea breath tests.
 Urea blood test:
   13C-urea blood test.
 Stool antigen test:
   Polyclonal stool antigen tests.
   Monoclonal stool antigen tests.
   Rapid stool antigen tests.

Invasive tests requiring endoscopy include the following:

 Biopsy urease testing (rapid urease test).
 Histology:
   Immunostaining.
   Fluorescent in situ hybridization (FISH).
   Molecular testing for susceptibility.
   Molecular tests for virulent factors (VacA-CagA).
 Brush cytology.
 Bacterial culture:
   Susceptibility tests.

The choice of test depends on clinical setting, local availability, and cost and use of medications (e.g., use of PPIs, bismuth, or antibiotics) that reduce the density of* H. pylori* and thus reduce the accuracy of tests for active infection. The presence of such potentially interfering agents is not an absolute contraindication for testing as testing can generally be delayed for a time during which those drugs are discontinued. The choice of a test is also influenced by the pretest probability of the infection [17].

### 5.1. Noninvasive Tests

#### 5.1.1. Serologic Tests

*H. pylori* infections are associated with a strong humoral immune response and the presence of serum IgG antibodies against* H. pylori* has been proven to provide a reliable assessment of current or previous infection. However, the presence of antibodies can remain for a long time after the infection; thus, a positive serologic test in a patient should not automatically imply the presence of an active infection. Most common serologic tests are based on an enzyme-linked immunosorbent assay (ELISA) technology. A meta-analysis of 21 studies with commercially available ELISA kits reported overall sensitivity and specificity of 85% and 79%, respectively [18]. Recently, several kits were evaluated in Europe and a number showed high sensitivity and susceptibility [19]. As a general rule, one should only use what has been validated locally or regionally. Although IgG, IgM, and IgA tests are commercially available, only the IgG tests are recommended as the others generally have poor reliability.

As with any test, prevalence of the* H. pylori* infection and the pretest probability influence the positive or negative predictive values [20, 21]. Overall, where the prevalence of* H. pylori* infection and the pretest probability are low, the negative predictive value of a serologic test is high whereas false positives are more frequent, with the opposite in high prevalence/high pretest probability cases (i.e., the positive predictive value is high but there is increased prevalence of false negative results). For example, a patient with a confirmed peptic ulcer would have a high pretest probability of infection such that it would be acceptable to initiate treatment based upon a positive serology, whereas a negative test would have a good chance of being false negative and should prompt confirmation using a test for active infection. In contrast, a negative test would have a good chance of being false negative and should prompt confirmation using a test for active infection. On the other hand, a positive serologic test in a patient with symptomatic gastroesophageal reflux from low prevalence regions would likely be a false positive and confirmation with a test for active infection would be prudent before initiation of therapy. Antibody testing cannot be used to confirm eradication. However, if a known positive antibody test becomes negative after many months, one can assume that it reliably predicts a successful outcome of therapy. It has been demonstrated that* H. pylori *titers declined by approximately 50% at 3 months, and seroconversion from detectable to undetectable levels at 18 months after therapy had a specificity of 100% proving to reliably correlate with cure [22]. However, the seroconversion does not occur often. Serologic testing might be useful where the pretest probability is high (e.g., active peptic ulcer) and tests for active infection are negative possibly because of the presence of factors that reduce the bacterial load such as antibiotic or bismuth use or widespread atrophy gastritis such as in gastric atrophy or MALT lymphoma.

A number of rapid office-based IgG kits, the so-called “near-patients tests,” have been developed. The more convenient ones use one drop of whole blood obtained by finger-prick; most of these tests have lower sensitivity and specificity than traditional ELISA tests and they are generally not recommended [23]. Although* H. pylori* vary in virulence, no clinical utility has been found in relation to assessing the presence of putative* H. pylori* virulence factors such as* CagA* or* VacA *[9].

#### 5.1.2. Saliva and Urine Tests

Antibody tests using saliva and urine have been developed because samples can be easily obtained especially from children. Studies indicate that IgG assays of saliva are not as sensitive as histology or serum testing [24, 25]. Generally, because of the low prevalence of infection in children, all tests will be associated with a high false positive rate and, as a rule of thumb, only children with two positive tests based on different methods should be considered to be* H. pylori *infected.

#### 5.1.3. Urea Breath Test (UBT)

The urea breath test is the noninvasive test of choice for the diagnosis of* H. pylori *[26, 27]. The method is based on* H. pylori*'s urease activity which splits urea into ammonia and carbon dioxide. The test can be performed with the urea labeled with radioactive isotope of carbon 14C or the nonradioactive naturally occurring stable isotope, 13C. The carbon-labeled urea is given orally, often in association with a test meal in order to delay gastric emptying and increase contact time with the mucosa. The preferred test meal is citric acid which also acidifies the stomach and inhibits non-*H. pylori* urease activity. The test is administered to the patient fasting from solid food for at least 1 hour.* H. pylori *urease liberates labeled CO_2_ that is detected in breath samples usually obtained 15 to 20 minutes after urea ingestion [26, 28]. The 14C-UBT requires a scintillation counter and technicians trained in the use of radioactive chemicals. The 13C-UBT requires a mass or infrared spectrometer. There are nuclear regulatory concerns for use of the 13C test in children or pregnant women. Generally, the use of radioisotopes should be restricted to those in need.

The UBT is a robust test with high sensitivity (95%) and specificity (95% to 100%) for the detection of active* H. pylori *infections although it is less accurate in children below 6 years of age unless one calculates the result using the urea hydrolysis rate [29]. False positive results are uncommon except in areas where atrophic gastritis is common and the test does not include citric acid [28, 30]. False negative results may be observed in patients who are taking antisecretory therapy, bismuth, or antibiotics and patients with upper gastrointestinal bleeding [31]. To reduce false negative results, the patient should be off antibiotics for at least four weeks and off PPIs for at least two weeks [9].

#### 5.1.4. 13C-Urea Blood Test

A blood version of the 13C-urea test (Ez-HBT, Metabolic Solutions Inc., Nashua, NH) was approved by the FDA as a noninvasive tool for diagnosis of* H. pylori *infection. This test is performed by measuring blood levels of 13C at baseline and 60 min after ingestion of 13C-urea. Although the test demonstrated excellent sensitivity of 92% to 100% and specificity of 96% to 97% [32], it is not used in clinical practice.

#### 5.1.5. Stool Antigen Tests

*H. pylori* present in the stomach are excreted in the stool. Available qualitative enzyme immunoassay commercial kits have been shown to be able to detect* H. pylori *protein antigens in a concentration of nanograms per mL of stool. Studies evaluating the ability of fecal antigen tests to diagnose* H. pylori *infection have generally been supportive. Polyclonal stool antigen testing has been proven to be less sensitive and specific than tests using monoclonal antibodies and is no longer recommended [9, 26, 33, 34]. The sensitivity and specificity reported for stool antigen tests based on monoclonal antibodies are similar to those of the urea breath test [7, 9] such that the tests can be used interchangeably. Both require the same precautions for the initial diagnosis of* H. pylori *infection and for confirming eradication following therapy [35]. For patients unable to stop PPI therapy two weeks prior to stool antigen testing, positive test results can be considered as true positive whereas negative results may represent false negatives and should be confirmed with repeat testing two weeks after stopping PPI therapy. For patients complaining of severe symptoms, antacids or histamine-2 receptor antagonists, which do not interfere with testing, are allowed [36]. Because of prolonged excretion of* H. pylori* antigens, it has been recommended that confirmation of cure testing be delayed until 6 weeks after the end of therapy.

#### 5.1.6. Rapid Stool Antigen Tests

A number of rapid (in the office)* H. pylori *stool antigen tests have been developed. Two large studies demonstrated high accuracy with pretreatment sensitivities of 93% and 95% and specificities of 89% and 87%. Following eradication, the reported sensitivities and specificities were 94% and 100%, 97% and 91%, respectively [37]. However, there are a number of other reports and abstracts showing much lower success with this and the use of rapid stool antigen test is not recommended [9].

### 5.2. Invasive Tests

Invasive tests typically require upper gastrointestinal endoscopy (EGD). EGD is a gold standard for epigastric symptoms because it allows direct inspection of the upper gastrointestinal mucosa and gives the opportunity to take biopsy samples. EGD is widely available in developed countries. However, it is expensive, unpleasant, time-consuming, and not without risk. Upper endoscopy is indicated in patients with alarm features (see the list of alarm or “red flags” prompting endoscopy for the evaluation of patients with dyspepsia) or those aged ≥55 years according to the American Gastroenterological Association and American College of Gastroenterology guidelines [2, 5, 38]. In Europe, the suggested age cut-off is 45 years for patients with persistent dyspepsia [9]. Biopsies of the stomach should be obtained to rule out* H. pylori* and for the histological evaluation of the gastric mucosa [9]. Specimens can also be used for bacterial culture and antibiotic susceptibility testing especially in patients who have previously failed* H. pylori* eradication. Other findings need to be treated according to the diagnosis.

#### 5.2.1. Biopsy Urease Testing

The biopsy urease activity often called rapid urease testing is based on the fact that* H. pylori* contain the urease enzyme and thus the presence of the infection can easily be identified using a colorimetric test based on the pH change when urea is hydrolyzed into ammonia and CO_2_. A number of gel-based, liquid-based, and paper-based tests are commercially available with similar diagnostic accuracy [39]. Some of the newer tests provide reliable data within one hour giving the gastroenterologist the possibility of providing* H. pylori* eradication treatment to the patients before leaving the endoscopic room [40]. Inexpensive and reliable homemade rapid urease test (urea broth plus one drop of 1% phenol red as a pH indicator) could be made in any laboratory. The sensitivity and specificity of biopsy urease tests are approximately 90% to 95% and 95% to 100%, respectively [38]. Recent gastrointestinal bleeding, use of PPIs and/or antibiotics and/or bismuth-containing compounds, and presence of atrophic gastritis and/or diffuse intestinal metaplasia may result in false negative test [30, 41]. On the base of experience,* H. pylori* is more frequently localized in the antrum and corpus (80%), only in the corpus in 10% and only in the antrum in 8% of cases [42].

Because a positive rapid urease test is based on the bacterial load in the gastric biopsy, when obtaining tissue samples from the antrum and the corpus, use of large forceps/or multiple samples increases the sensitivity of the test [21, 28, 43].

False positive tests are unusual; however, mouth flora may produce urease and contaminate samples. It is important for the endoscopist to take specimens from macroscopically normal mucosa as* H. pylori* colonize healthy gastric tissue and biopsies obtained from abnormally appearing mucosa (e.g., intestinal metaplasia) or from lesion margins are often negative.

#### 5.2.2. Histology

Gastric biopsies provide information regarding the presence and type of gastritis and whether it is complicated by intestinal metaplasia, dysplasia, atrophy, MALT lymphoma, or gastric cancer. Hematoxylin and Eosin (H&E) stain is excellent to define the gastric morphology but is poor for detecting* H. pylori* and a special stain is recommended such as a modified Giemsa (2% diluted).

The increasing use of PPIs which promote the presence of coccoid forms of* H. pylori* [44], on the gastric mucosa, has led many laboratories to abandon these nonspecific stains and instead use immunohistochemistry with specific anti-*H. pylori* antibodies for their final determination. Despite the high sensitivity of histology, problems related to sampling, handling, and processing the tissue specimens and interobserver variability among pathologists could affect results [45]. Because of the patchy colonization of bacteria, it is possible to increase the accuracy with multiple biopsies. In one study, the combination of four biopsy sites (lesser and greater curvature of the mid* antrum*, lesser and greater curvature of the mid body) was found to provide a good yield for the detection of* H. pylori *and the assessment of atrophic gastritis extent [45].

#### 5.2.3. Gastritis Assessment

Inflammatory cells in normal gastric mucosa are absent or rare. Because* H. pylori* infection results in marked infiltration of the mucosa with acute and chronic inflammatory cells, histology can indirectly point to the presence of the infection. The acute, or active, inflammatory component consists of neutrophils infiltrating the surface, foveolar epithelium, and the lamina propria. This is characteristically accompanied by a chronic inflammatory component consisting of lymphocytes, plasma cells, and scattered macrophages. This pattern is often called an acute-on-chronic gastritis (or active chronic gastritis) and is characteristic of* H. pylori* infections. Lymphoid follicles are often present.

Pathologists often use an organized scoring system to describe their findings (e.g., the Updated Sydney System) [46]. The Sydney System evaluates histology, topography, morphology, and aetiology and scores the histology using visual analogue scales (e.g., to score the density of* H. pylori*). The Sydney System approach is then used in systems to stage gastric cancer risk such as the OLGA (Operative Link for Gastritis Assessment) staging system [47].

*H. pylori *on the morphological analysis appears to the pathologist as typical spiral or curved shaped bacteria on the epithelial surface and in the mucus layer of the biopsy specimen. As noted above, the widespread use of PPIs often results in a few non-*H. pylori* bacteria or coccoid forms seen with special stains and has led many pathologists to always confirm that they are* H. pylori *by using immunohistochemical stains [48].

#### 5.2.4. Immunostaining Techniques

Immunohistochemistry using an immunoperoxidase technique following heat induced antigen retrieval for detecting* H. pylori *in gastric biopsy has been proven to be highly sensitive, easy to use, and reliable despite being expensive [48].

#### 5.2.5. Brush Cytology

In this technique, the mucosal surface is sampled using an endoscopic or even orally swallowed brush and then stained (e.g., with Quick Diff) for organisms and* H. pylori*. Brush cytology has a reported sensitivity of 95% to 98% and specificity of 96%, respectively [49].

#### 5.2.6. Molecular Tests

Polymerase chain reaction (PCR), in situ hybridization, and real-time PCR have also been used to detect* H. pylori, *assess antibiotic susceptibility, or evaluate the presence of putative virulence factors. The sensitivity and specificity for the diagnosis of* H. pylori *infection, using* in situ *hybridization with biotinylated probes, have been reported to be 95% to 100% [50]. Genomic DNA identified as targets for amplification are 16S rRNA,* ureA*,* ureB*,* ureC*,* fiaA*,* CagA*,* VacA*, and heat shock protein [51]. Real-time results can also be obtained shortening significantly the time for a result [52]. PCR is not routinely used for diagnosis because specificity has remained an issue and false positives are common probably related to as yet uncultured mouth flora.

PCR has proven useful for testing the susceptibility of* H. pylori *to clarithromycin and is based on the fact that clarithromycin resistance is related to point mutations (A-G transition) in the 23S rRNA [53]. PCR has also been used successfully on gastric biopsy specimens salvaged from rapid urease tests and even stool. Alternatively, fluorescence in situ hybridization (FISH) has been used on paraffin embedded gastric mucosal biopsies. The FISH method is rapid and accurate (92.6%) and would provide the clinician with important information regarding choice of therapy [54].

#### 5.2.7. Culture

The gold standard for the presence of most infectious diseases is culture of the organism. However, routine culture for* H. pylori *is not currently widely available and, more importantly, typically requires an invasive method (EGD) to obtain gastric samples. Any experienced microbiology laboratory can rapidly learn to culture* H. pylori* and the issues regarding transport to the microbiology laboratory are all easily overcome.

Bacterial growth is identified as* H. pylori *on the basis of colony morphology, cell morphology, Gram's stain, and positive biochemical reactions for catalase, urease, and oxidase. Experienced laboratories achieve 90% to 95% success.* H. pylori *have been isolated from stool but with poor overall success and stool culture is not currently practical [55].

#### 5.2.8. Susceptibility Testing

Culture is typically done to determine antibiotic susceptibility. Most laboratories use the epsilometer test (*E*-test) although agar dilution test is the reference method [56]. The *E*-test can accurately identify metronidazole susceptible strains but a reading of those resistant has been proven to be false in approximately 25% of cases. We recommend that all metronidazole resistant (by *E*-test) strains be confirmed by agar dilution. Given the current high prevalence of clarithromycin and fluoroquinolone resistance, it is prudent to have culture and antimicrobial susceptibility testing before using a clarithromycin-containing or fluoroquinolone-containing regimen as well as for deciding on therapy after initial treatment failure. However, there is strong argument among authors for pretreatment culturing and sensitivity testing after the first treatment failure and certainly after the second.

### 5.3. Treatment of* H. pylori* Infections

Eradication of* H. pylori* infection dramatically alters the natural history of gastritis and prevents its sequelae [6]. However,* H. pylori* infection is not easy to cure. As for other bacterial infections, antibiotics are necessary and, currently, a combination of antibiotics with antisecretory therapy is the standard of care. In addition, increasing antimicrobial resistance has made successful treatment of* H. pylori* infections a challenge as the effectiveness of many commonly recommended treatments such as traditional triple therapies has declined to unacceptable low levels [57]. The ideal therapeutic regimen should be based on antimicrobial susceptibility, but, in the real life, clinicians must choose the treatment without this approach. Therefore, the rules of thumb choosing the most appropriate treatment for patients are awareness about

1. antibiotics previously used by the patient and presence of drug allergy,
2. the rate of resistance against the most used antibiotics in the local area,
3. what works best locally.

In several cases, failure to obtain good results depends on clinician's unawareness of the poor results obtained locally with traditional therapies. Confirmation of eradication following treatment is mandatory [58]. Generally noninvasive tests such as stool antigen or urea breath tests should be used except where endoscopy is indicated because of the clinical findings. Confirmation of* H. pylori *eradication should be performed at least four weeks after treatment. This should be delayed if antibiotics are taken for reasons other than* H. pylori* eradication. Use of PPIs needs to be stopped, at least 2 weeks before testing, to reduce the chance of false negative results [36]. A positive UBT test done before this time is a reliable indication of treatment failure but negative tests should be confirmed.

Endoscopy with biopsy for culture might be indicated after several treatment failures to obtain specimens for culture and susceptibility testing. Antibody testing should not be used to confirm eradication since antibodies continue to be present for months or even years after eradication therapy.

## 6. Summary

For patients presenting with dyspeptic symptoms, the first goal is to separate those with organic causes (approximately 25%) from those with presumed functional dyspepsia (approximately 75%). A detailed history and physical examination are mandatory in order to confirm or exclude the presence of alarm symptoms (see the list of alarm or “red flags” prompting endoscopy for the evaluation of patients with dyspepsia). Patients with alarm features and/or above ≥45–55 years of age (based on the prevalence of gastric cancer in the specific geographical area) should undergo an early upper endoscopy and/or abdominal ultrasound according to the alarm features (Figure 1). In patients below 55 years of age with dyspeptic symptoms induced by NSAIDs, treatment should be discontinued and a trial of PPIs for eight weeks proposed. Patients with dyspeptic symptoms suggestive of delayed gastric emptying (early satiety, gastric fullness, and vomiting) could receive an empiric trial of prokinetic agents. In case of persistent symptoms, a study of gastric function (scintigraphy, breath testing, etc.) should be taken into account. Patients < 55 years of age without alarm features should be tested and treated for* H. pylori*, whatever the prevalence is in the region, especially those with a family history of peptic ulcer or gastric cancer. Noninvasive tests of choice are 13C-UBT or stool antigen assay. Eradication of* H. pylori* must be confirmed with noninvasive tests (13C-UBT or stool antigen assay). For patients positive for a family history of gastric cancer, to check eradication by EGD and biopsies is recommended. Endoscopic evaluation is indicated in patients with uninvestigated dyspepsia and without symptoms relief by empiric treatment or* H. pylori* eradication. Further evaluations should be oriented based on the patient's symptoms.
